# Changing from lipoprotein apheresis to evolocumab treatment lowers circulating levels of arachidonic acid and oxylipins

**DOI:** 10.1016/j.athplu.2024.01.005

**Published:** 2024-02-12

**Authors:** Chaoxuan Wang, Anne Kaufmann, Nadja Kampschulte, Ulf Elbelt, Ursula Kassner, Elisabeth Steinhagen-Thiessen, Anne Pietzner, Christoph Schmöcker, Dev Datta, Tiziana Sanpietro, Nils Helge Schebb, Karsten-H. Weylandt, Nadine Rohwer

**Affiliations:** a Medical Department B, Division of Hepatology, Gastroenterology, Oncology, Hematology, Palliative Care, Endocrinology and Diabetes, University Hospital Ruppin-Brandenburg, Brandenburg Medical School, Neuruppin, Germany; b Medical Department, Division of Psychosomatic Medicine, Campus Benjamin Franklin, Charité-Universitätsmedizin Berlin, Corporate Member of Freie Universität Berlin and Humboldt-Universität zu Berlin, Berlin, Germany; c Faculty of Health Sciences, Joint Faculty of the Brandenburg University of Technology Cottbus-Senftenberg, Brandenburg Medical School and University of Potsdam, Potsdam, Germany; d Chair of Food Chemistry, Faculty of Mathematics and Natural Sciences, University of Wuppertal, Wuppertal, Germany; e MVZ Endokrinologikum Berlin, Berlin, Germany; f Medical Department, Division of Hepatology and Gastroenterology (including Metabolic Diseases), Campus Virchow Klinikum, Charité-Universitätsmedizin, Corporate Member of Freie Universität Berlin and Humboldt-Universität zu Berlin, Berlin, Germany; g Department of Metabolic Medicine, University Hospital Llandough, Cardiff, United Kingdom; h Lipoapheresis Unit, Reference Center for Diagnosis and Treatment of Inherited Dyslipidemias, Fondazione Toscana Gabriele Monasterio, Pisa, Italy; i Department of Molecular Toxicology, German Institute of Human Nutrition Potsdam-Rehbruecke, Nuthetal, Germany

**Keywords:** Arachidonic acid, Cardiovascular disease, Evolocumab, Lipid mediator, Lipoprotein apheresis, Oxylipin, Polyunsaturated fatty acid

## Abstract

**Background and aims:**

Previous studies have shown that lipoprotein apheresis can modify the plasma lipidome and pro-inflammatory and pro-thrombotic lipid mediators. This has not been examined for treatment with protein convertase subtilisin/kexin type 9 inhibitors such as evolocumab, which are increasingly used instead of lipoprotein apheresis in treatment-resistant familial hypercholesterolemia. The aim of this study was to compare the effects of evolocumab treatment and lipoprotein apheresis on the fatty acid profile and on formation of lipid mediators in blood samples.

**Methods:**

We analyzed blood samples from 37 patients receiving either lipoprotein apheresis or evolocumab treatment as part of a previous study. Patients were stratified according to receiving lipoprotein apheresis (n = 19) and evolocumab treatment (n = 18). Serum fatty acid analysis was performed using gas chromatography flame ionization detection and plasma oxylipin analysis was done using liquid chromatography tandem mass spectrometry.

**Results:**

Changing from lipoprotein apheresis to evolocumab treatment led to lower levels of omega-6 polyunsaturated fatty acid (n-6 PUFA) including arachidonic acid, dihomo-γ-linolenic acid and linoleic acid. Moreover, several n-6 PUFA-derived oxylipins were reduced after evolocumab treatment.

**Conclusions:**

Given that arachidonic acid, either directly or as a precursor, is associated with the development of inflammation and atherosclerosis, evolocumab-mediated reductions of arachidonic acid and its metabolites might have an additional beneficial effect to lower cardiovascular risk.

## Highlights

• Changing from lipoprotein apheresis to evolocumab lowers blood arachidonic acid.
• Omega-3 fatty acid levels were not changed by evolocumab.
• Evolocumab was associated with a reduction in arachidonic acid-derived oxylipins.

## 1 Introduction

Lipoprotein apheresis is an effective therapeutic method for lowering blood low-density lipoprotein cholesterol (LDL-C) and lipoprotein(a) (Lp(a)) concentrations and to improve cardiovascular outcome in patients with atherosclerotic disease and therapy-refractory hypercholesterolemia [[1], [2], [3]]. However, lipoprotein apheresis is a time and cost intensive procedure and patients may benefit from alternative treatment options to reduce LDL-C that are simpler and more accessible. In recent years the introduction of protein convertase subtilisin/kexin type 9 (PSCK9) inhibitors has established a new treatment option to aggressively lower LDL-C and cardiovascular risk. Evolocumab is a human monoclonal immunoglobulin G2 that inhibits specifically human PSCK9 and prevents it from binding to the LDL receptor, thereby reducing LDL-C [4]. In a randomized double-blind placebo-controlled trial, patients treated with evolocumab had significantly lowered LDL-C levels and a reduced risk of cardiovascular events [5]. Furthermore, Baum et al. evaluated whether evolocumab can reduce the requirement for lipoprotein apheresis among patients currently undergoing a stable apheresis regimen (DE LAVAL study [6]). The authors found that evolocumab was able to reduce the need for lipoprotein apheresis in patients previously receiving regular weekly or biweekly lipoprotein apheresis. In addition, LDL-C, non-high-density lipoprotein cholesterol (non-HDL-C) and total cholesterol/HDL-C ratio significantly decreased in patients treated with evolocumab, whereas these parameters were stable in patients receiving lipoprotein apheresis [6].

Previous studies in the 1990s indicated, that lipoprotein apheresis is also able to decrease membrane-bound arachidonic acid levels in red blood cells and platelets which might decrease pro-inflammatory mediator formation such as thromboxanes [7,8].

In contrast, lipoprotein apheresis has been shown to increase the formation of circulating pro-inflammatory and pro-thrombotic lipid mediators in our previous studies [9,10]. Since activation of lipoxygenases and formation of arachidonic acid (AA, 20:4 n-6)-derived lipid mediators have been implicated in the development of inflammation and atherosclerosis [11], these observations might have consequences for the inflammatory state and cardiovascular risk associated with polyunsaturated fatty acid (PUFA)-derived lipid mediators in apheresis-treated patients [12]. In a first study with a small number of patients treated with heparin-induced extracorporeal low-density lipoprotein precipitation (HELP) apheresis we found significantly decreased levels of plasma omega-3 (n-3) PUFA as well as a trend towards an increase of autoxidative or 5- and 12-lipoxygenase lipid mediator formation in patients undergoing lipoprotein apheresis [10]. This was supported in a larger study, in which particularly HELP treatment was found to lead to decreases of essential n-6 and n-3 PUFA in blood plasma but significant increases of PUFA-derived autoxidative and lipoxygenase (LOX)-, as well as cyclooxygenase (COX)- and cytochrome P450 (CYP450)-derived oxylipins in direct pre-versus post-apheresis measurements [9].

N-6 PUFA AA as well as the n-3 PUFA eicosapentaenoic acid (EPA, 20:5 n-3) and docosahexaenoic acid (DHA, 22:6 n-3) can be processed by different enzymatic (e.g., COX, LOX, CYP450) and non-enzymatic (autooxidation) pathways, resulting in a wide spectrum of oxylipins of which several act as active lipid mediators. In addition to LDL-C-lowering therapy, n-3 PUFA such as EPA have been shown to reduce cardiovascular risk [13,14] and n-3 PUFA-derived lipid mediators have cardioprotective effects [15]. This is in contrast to many AA products from these enzymatic pathways that play a key role in many inflammatory diseases as pro-inflammatory mediators [16].

Against this background, the aim of the present study was to compare the effects of evolocumab treatment and lipoprotein apheresis on the fatty acid (FA) profile as well as on the formation of lipid mediators in blood samples from the study performed by Baum et al. [6]. We were able to show that treatment with evolocumab was associated with decreases of n-6 PUFA levels including AA, dihomo-γ-linolenic acid (DGLA) and linoleic acid (LA) as well as several AA- and LA-derived lipid mediators.

## 2 Patients and methods

### 2.1 Study population

Patients who received regular lipoprotein apheresis for LDL-C lowering for at least 3 months immediately before study screening and had no change to a stable weekly or every-2-week schedule or lipoprotein apheresis type for the most recent 4 weeks were included in the study. Patients were required to receive background pharmacological lipid-lowering therapy that included a high- or moderate-intensity statin dose, unless not tolerated. Pre-apheresis LDL-C concentration between ≥2.6 mmol/L (100 mg/dL) and ≤4.9 mmol/L (190 mg/dL) were accepted for enrollment. All locally approved lipoprotein apheresis types were included. Patients with homozygous familial hypercholesterolemia (FH) were excluded. The study was approved by each institutional review board and all procedures were conducted in accordance with the Code of Ethics of the World Medical Association (Declaration of Helsinki). All patients provided written informed consent. Qualified researchers may request data from Amgen clinical studies [6].

### 2.2 Study design

This study is following up on a randomized, active controlled, open-label, multicenter, parallel-group trial conducted at 15 centers in Australia, Europe, and the United States between December 2015 and January 2017 [6]. Patients were randomized 1:1 to continue receiving lipoprotein apheresis or to discontinue and start treatment with evolocumab 140 mg subcutaneously every-2-week for the next 6 weeks of the study. Randomization was stratified by pre-apheresis LDL-C level at screening (<4.1 mmol/L [160 mg/dL] vs ≥ 4.1 mmol/L). Patients remained on the same background lipid-lowering regimen received in the 4 weeks before entering the study [6]. Lipid profile parameters including Lp(a), triglycerides, total cholesterol, LDL-C, HDL-C and very low-density lipoprotein cholesterol (VLDL-C) were determined before lipoprotein apheresis or evolocumab at day 1 (baseline), week 4 and 6 [6]. Fatty acid and lipid mediator concentrations were measured from serum and plasma samples, respectively, at day 1 and week 6 as described below. Some patient samples were excluded from fatty acid and lipid mediator measurements as the patients had undergone lipoprotein apheresis during evolocumab treatment or due to improper sample storage. The precise number of patient samples used in the analysis is specified in the figure legends.

### 2.3 Fatty acid measurement

100 μl of serum per sample was used for the gas chromatography (GC) preparation. Methylation and extraction of FA were carried out on the basis of an established protocol [17]. Briefly, frozen samples were thawed at room temperature. All samples were then mixed with 50 μl pentadecanoic acid (PDA, 1 mg/ml, Merck Schuchardt OHG, Hohenbrunn, Germany) as internal standard, 500 μl borontrifluoride (BF3, Sigma-Aldrich Chemie GmbH, Taufkirchen, Germany) in 14% methanol (Merck KgaA, Darmstadt, Germany), and 500 μl n-hexane (Merck KgaA, Darmstadt, Germany) in glass vials and tightly closed. After vortexing, samples were incubated for 60 min in a preheated block at 100 °C. After cooling down to room temperature, the mixture was added to 750 μl water, vortexed, and extracted for 4 min. Then all samples were centrifuged for 5 min (RT, 3500 rpm). From each sample, 100 μl of the upper n-hexane layer was transferred into a micro-insert (placed in a GC glass vial), tightly closed and analyzed by GC.

GC was performed on a 7890B GC System (Agilent Technologies, Santa Clara, USA) with a HP88 Column (112/8867, 60 m x 0,25 mm x 0,2 μm, Agilent Technologies, Santa Clara, United States) with the following temperature gradient: 50 °C–150 °C with 20 °C/min, 150 °C–240 °C with 6 °C/min and 240 °C for 10 min (total run time 30 min). Nitrogen was used as carrier gas (constant flow 1 ml/min). 1 μl of each sample was injected into the injector (splitless injection, 280 °C). The flame ionization detector (FID) analysis was performed at 250 °C with the following gas flows: hydrogen 20 ml/min, air 400 ml/min, make up 25 ml/min. Methylated FA in the samples were identified by comparing the retention times with those of known methylated FA of the Supelco® 37 FAME MIX standard (CRM47885, Sigma Aldrich, Laramie, USA) and single FAME standards purchased from Cayman Chemicals (Ann Arbor, MI, USA). Analysis and integration of the peaks were carried out with OpenLAB CDS ChemStation Edition (Agilent Technologies, Santa Clara, USA). The concentrations of PUFAs in the samples were calculated in relation to the known concentration of the internal standard PDA. For the study, 16 FA were included as follows: myristic acid (C14:0), palmitic acid (C16:0), stearic acid (C18:0), arachidic acid (C20:0), behenic acid (C22:0), lignoceric acid (C24:0), palmitoleic acid (C16:1n7c), oleic acid (C18:1n9c), nervonic acid (C24:1n9), eicosapentaenoic acid (EPA, C20:5n3), docosapentaenoic acid (DPA, C22:5n3), docosahexaenoic acid (DHA, C22:6n3), linoleic acid (LA, C18: 2n6), dihomo-gamma-linolenic acid (DGLA, 20:3n6), arachidonic acid (AA, 20:4n6), adrenic acid (AdA, C22:4n6).

### 2.4 Lipid mediator measurement

Analysis of free eicosanoids and other oxylipins was carried out as described before [[18], [19], [20]]. In brief, 500 μl plasma was used, and 10 μl antioxidant mixture (0.2 mg/ml BHT, 100 μM sEH inhibitor and 100 μM COX inhibitor) and 10 μl of a mixture of deuterium labeled internal standards (IS) (100 nM in MeOH) were added. Proteins were precipitated by addition of 1400 μl MeOH and freezing the samples at −80 °C overnight. After centrifugation, the supernatant was diluted with 0.1 M disodium hydrogen phosphate buffer yielding a MeOH content <17% (pH 6) and loaded on a preconditioned SPE cartridge (Bond Elut Certify II, 200 mg, 3 mL; Agilent, Waldbronn, Germany). The SPE procedure was performed as described. Reconstituted samples were analyzed by targeted LC-MS/MS following negative electrospray ionization in scheduled selected reaction monitoring mode. Quantification was carried out based on analyte to corresponding IS area ratio using external calibration with least squares regression (1/x^2^ weighting).

### 2.5 Statistical analysis

Statistical analysis was done using GraphPad Prism software (San Diego, California, USA). Outliers were identified using the Grubbs outlier test. Comparisons between two groups of normally distributed data with equal variances were performed using the unpaired two-tailed Student's *t*-test, for serial comparisons of normally distributed data with equal variances the paired two-tailed Student's *t*-test was used. To correct for multiple comparisons performed, we used Bonferroni correction. The significance levels after Bonferroni correction are given in the figure legends. Sample size, statistical tests and *p* values are indicated in the figure legends or in the result section. Data are expressed as means + SEM.

## 3 Results

The effect of lipoprotein apheresis and evolocumab treatment on lipid profile parameters in these patients was described previously [6]. Baseline characteristics and changes in lipid parameters from baseline (day 1) to week 6 for the patients included in this substudy are summarized in Supplementary Table S1 and Supplementary Fig. S1. Baseline lipid levels at day 1 were not significantly different between the two treatment groups (Supplementary Table S1). Compared to lipoprotein apheresis, evolocumab-treated patients had lower Lp(a) (*p* = 0.0251) and total cholesterol (*p* < 0.001) values (Supplementary Fig. S1A) as well as lower values for LDL-C (*p* < 0.001) and VLDL-C (*p* = 0.0401) following the 6-week treatment (Supplementary Fig. S1B).

### 3.1 Effect of evolocumab treatment on fatty acid levels

**Fig. 1:**
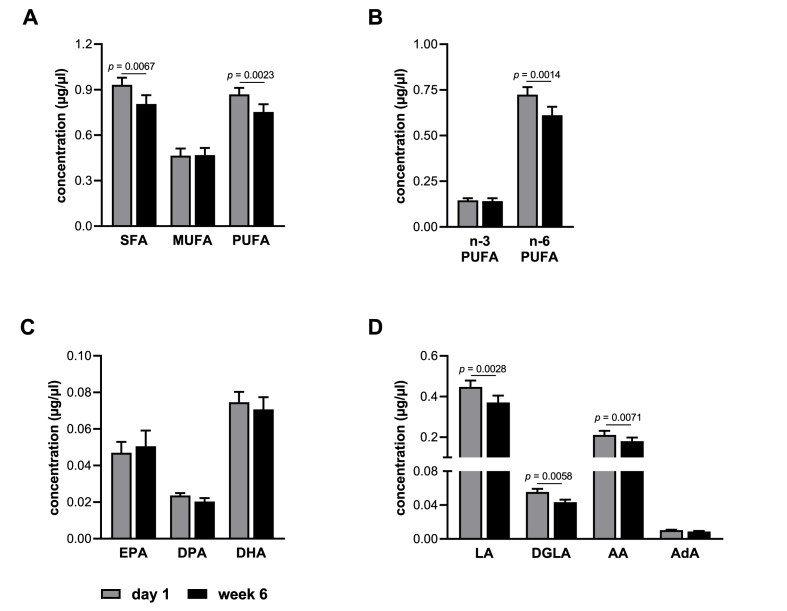
Fatty acid profile in the serum of patients receiving evolocumab. Shown are the concentrations at baseline (day 1) and after six weeks of treatment (week 6) of (A) SFA, MUFA and PUFA, (B) n-3 and n-6 PUFAs, (C) individual n-3 PUFAs EPA, DPA and DHA, and (D) individual n-6 PUFAs LA, DGLA, AA and AdA (n = 14). Values are presented as mean + SEM. Statistical analyses were performed by two-tailed paired Student's *t*-test. *P* < 0.0042 after Bonferroni correction is considered statistically significant.

To investigate the effect of evolocumab treatment on the FA profile, we analyzed a broad range of FA in the serum of 16 patients receiving evolocumab for six weeks by GC. Fig. 1 shows the FA profile grouped as saturated fatty acids (SFA), monounsaturated fatty acids (MUFA) and PUFA as well as absolute concentrations of selected n-3 and n-6 PUFA. The FA profile is characterized by relatively high n-6 PUFA and low n-3 PUFA levels (Fig. 1). It is well established that the FA composition is closely related to nutrition and dietary habits. Low n-3 PUFA levels, as observed in this study, are usually found in populations that are adapted to industrial based or western diets, such as in Western Europe and North America [21].

Treatment with evolocumab for six weeks was associated with a reduction by approximately 14% of both SFA (*p* = 0.0067) and PUFA (*p* = 0.0023, Fig. 1A). N-3 PUFA levels were not changed by evolocumab treatment, while n-6 PUFA levels were significantly reduced by 16% (*p* = 0.0014, Fig. 1B). The most abundant n-6 PUFA in human serum samples was LA (Fig. 1D). AA levels were half that of LA, but still 3- to 4-fold higher than of the n-3 PUFA EPA and DHA (Fig. 1C + D). In line with the significant reduction of n-6 PUFA, evolocumab treatment reduced specific n-6 PUFA, namely LA, DGLA and AA (Fig. 1D). In contrast to the decreases of the previously mentioned n-6 PUFA, changes in the n-3 PUFA EPA, docosapentaenoic acid (DPA) and DHA by evolocumab treatment were not observed (Fig. 1C).

Taken together, these data suggest that evolocumab treatment exhibited a potent fatty acid lowering effect, particularly for n-6 PUFA, while having no effect on n-3 PUFA.

### 3.2 Comparison of the effects of evolocumab treatment and lipoprotein apheresis on fatty acid levels

Next, we aimed to compare the effects of the two lipid-lowering therapies on the FA profiles after six weeks of therapy. Blood samples were taken at day 1 (baseline) and week 6. It should be noted that all patients received lipoprotein apheresis for at least three months before day 1. Baseline FA concentrations at day 1 were not significantly different between the two treatment groups (Supplementary Table S1). Supplementary Fig. S2 compares the FA profile as well as the absolute concentrations of selected n-3 and n-6 PUFA of patients that received lipoprotein apheresis or evolocumab at week 6. Treatment with evolocumab led to a decrease of SFA (*p* = 0.0263) compared to lipoprotein apheresis (Supplementary Fig. S2A). Concentrations of PUFA and n-6 PUFA in serum of evolocumab-treated patients were reduced, yet not significantly, compared to lipoprotein apheresis patients (Supplementary Fig. S2A + B). No significant differences were found for n-3 PUFA as a whole or for the individual n-3 PUFA EPA, DPA and DHA between the two groups (Supplementary Fig. S2B + C). With respect to individual n-6 PUFA, the absolute amounts of LA, DGLA and AA were lower under evolocumab treatment (Supplementary Fig. S2D).

**Fig. 2:**
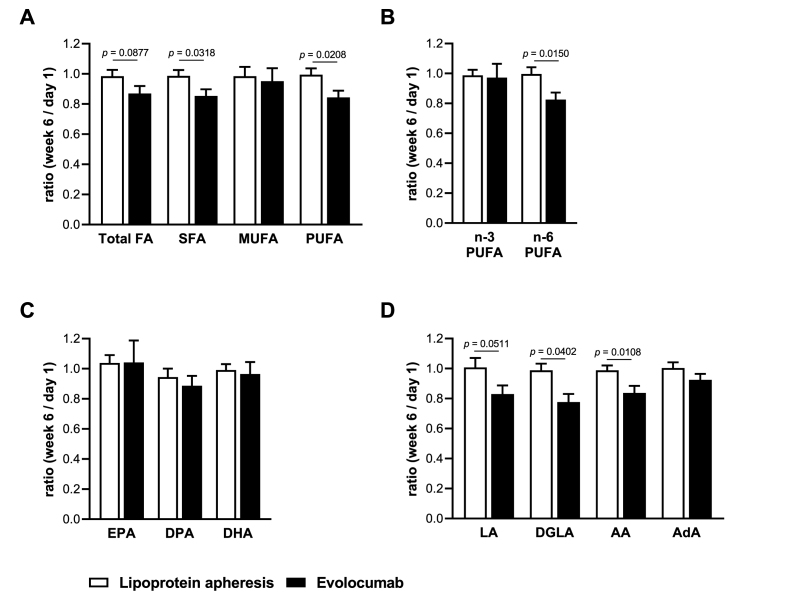
Change of the fatty acid concentrations of patients receiving lipoprotein apheresis or evolocumab after six weeks of treatment. Shown are the mean ratios of the fatty acid concentration at week 6 to the baseline concentration at day 1 of (A) total FA, SFA, MUFA and PUFA, (B) n-3 and n-6 PUFAs, (C) individual n-3 PUFAs EPA, DPA and DHA, and (D) individual n-6 PUFAs LA, DGLA, AA and AdA (n = 18 for lipoprotein apheresis, n = 14 for evolocumab). Values are presented as mean + SEM. Statistical analyses were performed by two-tailed unpaired Student's *t*-test. *P* < 0.0039 after Bonferroni correction is considered statistically significant.

To further elucidate the effects of lipoprotein apheresis and evolocumab on the fatty acid profile, the week 6/day 1 ratio was calculated by dividing the absolute concentration of the FA of interest at week 6 by the absolute concentration on day 1 (Fig. 2). Ratios between week 6 and day 1 in the apheresis group were close to 1 for all FA, indicating no marked changes in FA concentrations from day 1 until week 6, as expected given that apheresis treatment was basically continued as before day 1 (Fig. 2). In contrast, the ratios in the evolocumab treatment group were below 1 for total FA, SFA, PUFA, n-6 PUFA and all analyzed individual n-6 PUFA, indicating a reduction in concentrations of the mentioned FA due to evolocumab treatment. Comparing the ratios of both groups, a marked difference was found for SFA (*p* = 0.0318), PUFA (*p* = 0.0208) and n-6 PUFA (*p* = 0.0150), as well as for the n-6 PUFA LA (*p* = 0.0511) DGLA (*p* = 0.0402) and AA (*p* = 0.0108, Fig. 2).

Overall, these results suggest that in comparison to lipoprotein apheresis, evolocumab treatment markedly lowers the levels of n-6 PUFA as a whole as well as the individual n-6 PUFA LA, DGLA and AA.

### 3.3 Effect of evolocumab treatment on levels of monohydroxy fatty acids

In order to investigate the effect of evolocumab on oxylipin profiles, we performed LC-MS/MS analysis with plasma samples from 14 patients treated with evolocumab for six weeks. We focused our analysis on monohydroxy fatty acids as they reflect the LOX, COX and autoxidation pathway of the AA cascade and act, either directly or as precursors, as mediators in the context of inflammatory processes and cardiovascular diseases.

**Fig. 3:**
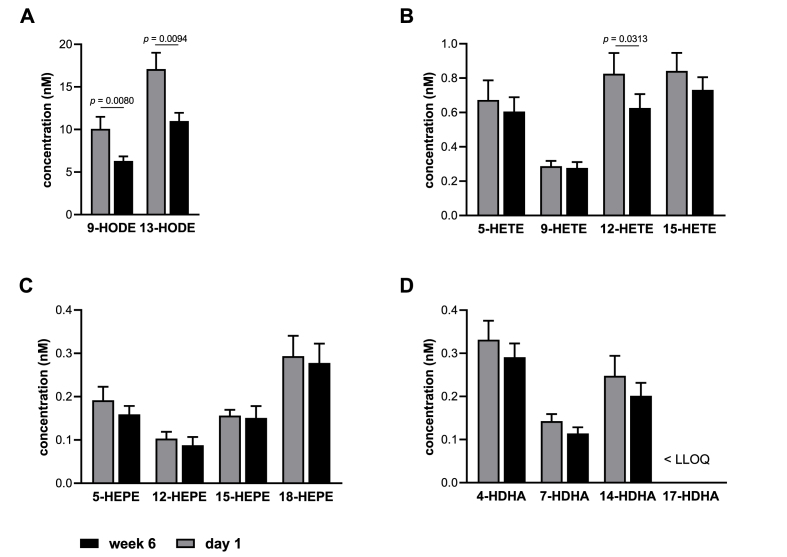
Oxylipin levels in the plasma of patients receiving evolocumab. Shown are the concentrations at baseline (day 1) and after six weeks of treatment (week 6) of selected monohydroxy fatty acids derived from (A) LA, (B) AA, (C) EPA, and (D) DHA. Values are presented as mean + SEM (n = 14). Statistical analyses were performed by two-tailed paired Student's *t*-test. *P* < 0.0039 after Bonferroni correction is considered statistically significant. LLOQ, lower limit of quantification.

As can be seen in Fig. 3, the most abundant monohydroxy fatty acids in human plasma were LA-derived 9- und 13-Hydroxyoctadecadienic acids (HODE) followed by the AA-derived oxylipins 15-Hydroxyeicosatetraenoic acid (HETE), 12-HETE, 5-HETE and 9-HETE. In line with the decreased concentrations of LA and AA (Fig. 1D), 9-HODE (*p* = 0.0080), 13-HODE (*p* = 0.0094) and 12-HETE (*p* = 0.0313) were markedly reduced after six-week treatment with evolocumab (Fig. 3A + B). 5-HETE and 15-HETE also showed a trend to be lower. Consistent with the low n-3 PUFA status of the subjects, concentrations of EPA and DHA metabolites were lower than concentrations of AA oxylipins and significant changes in EPA and DHA metabolites due to evolocumab treatment were not observed (Fig. 3C + D).

In summary, these results show that six-week evolocumab treatment was associated with a marked reduction in LA- and AA-derived monohydroxy fatty acid levels.

### 3.4 Comparison of the effects of evolocumab treatment and lipoprotein apheresis on monohydroxy fatty acids

We also compared effects of the two lipid-lowering therapies on the profile of monohydroxy fatty acids after six weeks of therapy. As can be seen from Supplementary Fig. S3, no significant differences were found between continued lipoprotein apheresis versus evolocumab treatment group. Solely, 9-HODE (*p* = 0.0754) showed a trend towards a lower concentration in the evolocumab group compared to the lipoprotein apheresis group, however the difference was not statistically significant (Supplementary Fig. S3A).

**Fig. 4:**
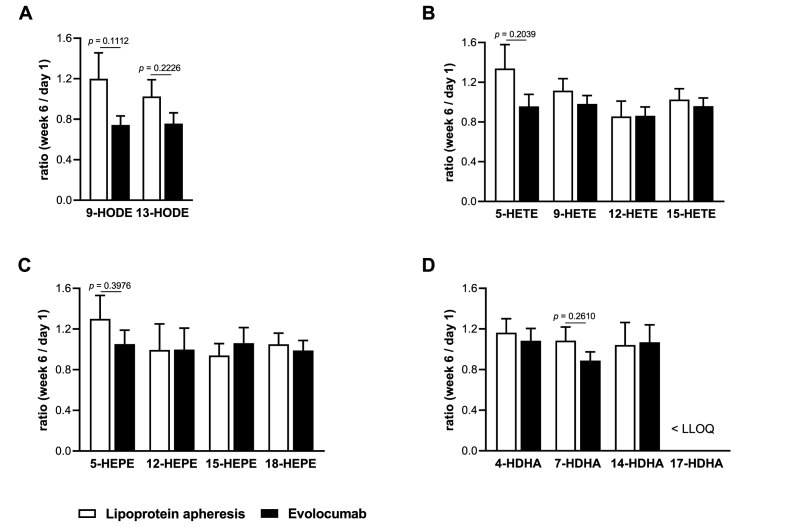
Change of the oxylipin concentrations in the plasma of patients receiving lipoprotein apheresis or evolocumab. Shown are the mean ratios of the oxylipin concentration at week 6 to the baseline concentration at day 1 of selected monohydroxy fatty acids derived from (A) LA, (B) AA, (C) EPA, and (D) DHA. Values are presented as mean + SEM (n = 18 for lipoprotein apheresis, n = 14 for evolocumab). Statistical analyses were performed by two-tailed unpaired Student's *t*-test. *P* < 0.0039 after Bonferroni correction is considered statistically significant. LLOQ, lower limit of quantification.

To better assess putative differences between the two lipid-lowering treatment approaches, we again compared the week 6 to day 1 ratios. As shown before when comparing the absolute concentrations, there were no significant differences between the week 6 to day 1 ratios of the evolocumab and the apheresis group (Fig. 4). However, evolocumab treatment showed a trend for a stronger reduction in the concentrations of 5-LOX-derived hydroxy-PUFA 5-HETE (*p* = 0.2039), 5-Hydroxyeicosapentaenoic acid (HEPE, *p* = 0.3976) and 7-Hydroxydocosahexaenoic acid (HDHA, *p* = 0.2610) in comparison to lipoprotein apheresis (Fig. 4B – D). Moreover, LA metabolites 9-HODE (*p* = 0.1112) and 13-HODE (*p* = 0.2226) also showed a trend towards a decrease in the evolocumab group when compared to the lipoprotein apheresis group (Fig. 4A).

## 4 Discussion

The present study examined differences in fatty acid profiles and oxylipin levels in human blood samples from individuals receiving lipoprotein apheresis or evolocumab, two treatment options to lower LDL-C and cardiovascular risk in patients with atherosclerotic disease and therapy-refractory hypercholesterolemia.

Using GC to analyze the fatty acid profile, we observed that treatment with evolocumab markedly reduced n-6 PUFA levels, particularly LA, DGLA and AA. In contrast, levels of n-3 PUFA remained unchanged by evolocumab treatment. A wealth of data indicated that n-6 PUFA metabolic pathways are involved in a variety of inflammatory processes, while n-6 PUFA are precursors of pro-inflammatory lipid mediator [15]. For example, AA, one of the most important n-6 PUFA in mammals, is the precursor for prostaglandins and leukotrienes, which critically contribute to inflammation [15]. Contrary to this, n-3 PUFA EPA and DHA can reduce the inflammatory response by interfering with the arachidonic acid metabolism [22]. There is growing evidence that the unbalanced intake of n-6 PUFA and n-3 PUFA changes the physiological state to a more pro-inflammatory and thrombotic state, causing vasospasm, vasoconstriction and increased blood viscosity, as well as the development of diseases associated with these conditions [23]. Chronic inflammation increases the risk of atherosclerosis and insulin resistance, which are the main mechanisms for the development of cardiovascular disease [24].

Lipoprotein apheresis is known to reduce markers of vascular inflammation in the plasma and can also reduce levels within the plaque which results in plaque stabilization [[25], [26], [27]]. Furthermore, given that lipoprotein apheresis – in contrast to evolocumab treatment – has strong effects on Lp(a), it can be employed to substantially lower risk of atherosclerosis progression in patients with increased Lp(a) [28].

Our results provide evidence that lipoprotein apheresis and evolocumab treatment might affect n-6 and n-3 PUFA profiles differentially. Compared to lipoprotein apheresis, evolocumab reduces n-6 PUFA levels significantly. We hypothesized that evolocumab treatment thus has the potential to reduce the inflammatory response by lowering AA levels, thereby having additional benefits besides lowering of lipoproteins. Due to the effect on n-6 PUFA, we hypothesized that evolocumab might also interfere with formation of n-6 PUFA-derived lipid mediators. By use of LC-MS/MS analysis, we observed a marked decrease of LA-derived 9-HODE and 13-HODE as well as AA-derived 12-HETE after six-week treatment with evolocumab.

12-HETE is a bioactive lipid metabolite of AA and mainly produced by platelets via the 12-lipoxygenase pathway. High levels of 12-HETE have been described in several diseases such as arterial hypertension, diabetes mellitus and atherosclerosis and suggest its involvement in these pathological conditions [[29], [30], [31]]. The presence of 12-HETE in atherosclerotic plaques has been demonstrated in aortic samples obtained from atherosclerotic rabbits [32,33]. More recently, higher 12-HETE levels have been observed in patients with acute coronary syndrome and coronary artery disease [34,35]. Moreover, several studies have been published which suggested explanations for the underlying mechanism of the connection between 12-HETE and the development of atherosclerosis. It has been suggested that 12-HETE exerts pro-atherogenic effects due to its potential role on endothelial cell dysfunction and monocyte recruitment [[36], [37], [38]]. In addition, 12-HETE downregulates macrophage efferocytosis, an important anti-inflammatory function of macrophages that limits atherosclerotic plaque progression [34]. These findings indicate that 12-HETE exhibits pro-inflammatory and pro-atherogenic properties and contributes to the development of atherosclerosis. In turn, treatment with evolocumab might thus limit atherosclerosis and lower the risk of cardiovascular events by decreasing 12-HETE production.

As early as two decades ago, Kühn and colleagues described the accumulation of HODEs in human atherosclerotic plaques [39]. Since then, the presence of 9- and 13-HODE in different stages of human atherosclerotic lesions has been confirmed repeatedly [40,41]. 9- and 13-HODE are stable oxidation products of LA, the most abundant fatty acid in atherosclerotic plaques, and have emerged as important indicators for oxidative stress [42]. However, with respect to atherosclerosis, particularly 13-HODE was described to have both pro- and anti-atherogenic effects [37]. Furthermore, there is accumulating evidence that 9- and 13-HODE have distinct biological properties depending on the stage of atherosclerosis [42]. In incipient atherosclerotic lesions, 13-HODE is the predominant form and activates protective/anti-inflammatory mechanisms which result in increased clearance of lipids and cell debris from the vascular wall. In advanced atherosclerotic lesions, 9-HODE is at least as abundant as 13-HODE and the net effect of both HODEs may be mainly harmful rather than beneficial. At this stage of disease, increased HODE levels thus contribute to atherosclerosis progression and cardiovascular risk. Since evolocumab treatment is primarily used in patients with an advanced atherosclerotic disease, it can be assumed that the evolocumab-mediated decrease of 9- and 13-HODE has a beneficial effect on the atherosclerotic process.

There are some limitations to the present study. One limitation is that all patients received lipoprotein apheresis for at least three months before the first blood sampling at day 1. Patients in the evolocumab group thus started treatment after the first blood drawing at day 1 on the basis of a preceding apheresis treatment, while apheresis patients basically continued their regular apheresis protocols. We observed marked differences within the evolocumab group when we compared the fatty acid and oxylipin levels at a baseline defined by the preceding apheresis treatment versus after six weeks of evolocumab therapy. Moreover, the study did not collect details of lipoprotein apheresis procedures which may have differed at the study sites because all locally approved and established apheresis types were accepted. Therefore, different forms of apheresis therapy were combined in one group. The effects of the individual apheresis methods on oxylipins activation may well differ as described by us before [9]. Another limitation of this study is that we did not account for differences in nutrition and PUFA intake. Furthermore, we did not analyze acute effects of evolocumab or apheresis treatment on fatty acid and oxylipin concentrations. Future studies will have to address these questions and determine the distribution of essential fatty acids and lipid mediators in a shorter period after therapy start.

In summary, we found that evolocumab treatment led to decreased serum levels of n-6 PUFA but has no effect on n-3 PUFA when compared to long-term apheresis treatment. This is in contrast to one of our earlier studies where we observed in patients that underwent lipoprotein apheresis a decrease of both n-3 and n-6 PUFA. However, in this previous study we assessed PUFA levels directly before versus directly after an individual apheresis session. We assume that evolocumab treatment has the potential to reduce the inflammatory response by lowering n-6 PUFA and in particular AA levels. Interestingly, treatment with evolocumab resulted in a significant decrease in n-6 PUFA and, consistent with this, there was also a trend towards a reduction in AA-derived 12-HETE and LA-derived 9- and 13-HODE. Given that all three of them have been implicated in atherosclerosis, we hypothesize that evolocumab might impede the atherosclerotic process and the risk of cardiovascular events by decreasing these pro-inflammatory and potentially pro-atherogenic lipid mediators.

## Author contributions

K.-H.W., N.H.S. and N.R. conceptualized and designed the study. A.K., A.P., C.W. and N.K. performed the fatty acid and oxylipin analyses. D.D and T.S. provided samples. A.P., C.S., C.W., N.K., N.R., U.E. and U.K. analyzed and interpreted the data. C.W., K.-H.W. and N.R. drafted the manuscript. A.P., C.S., D.D, E.S.-T., N.H.S, T.S., U.E. and U.K. critically revised the manuscript. K.-H.W. and N.R. supervised the study.

## Declaration of competing interest

The authors declare the following financial interests/personal relationships which may be considered as potential competing interests:

This study was supported by Amgen GmbH (Karsten-Henrich Weylandt). The remaining authors disclose no conflicts.

## References

[1] Grutzmacher P., Kleinert C., Dorbath C., Ohm B. (2015). Indications for apheresis as an ultima ratio treatment of refractory hyperlipidemias. Clinical research in cardiology supplements.

[2] Safarova M.S., Ezhov M.V., Afanasieva O.I., Matchin Y.G., Atanesyan R.V. (2013). Effect of specific lipoprotein(a) apheresis on coronary atherosclerosis regression assessed by quantitative coronary angiography, Atherosclerosis. Supplement.

[3] Schettler V.J.J., Neumann C.L., Peter C., Zimmermann T., Julius U. (2019). Lipoprotein apheresis is an optimal therapeutic option to reduce increased Lp(a) levels. Clinical research in cardiology supplements.

[4] Markham A. (2015). Evolocumab: first global approval. Drugs.

[5] Sabatine M.S., Giugliano R.P., Keech A.C., Honarpour N., Wiviott S.D. (2017). Evolocumab and clinical outcomes in patients with cardiovascular disease. N Engl J Med.

[6] Baum S.J., Sampietro T., Datta D., Moriarty P.M., Knusel B. (2019). Effect of evolocumab on lipoprotein apheresis requirement and lipid levels: results of the randomized, controlled, open-label DE LAVAL study. J Clin Lipidol.

[7] Brautigam R., Brautigam C., Lorenz R., Richter W.O., Engelmann B. (1997). Arachidonic acid of platelet phospholipids is decreased after extracorporeal removal of plasma low density lipoproteins in patients with familial hypercholesterolemia. Atherosclerosis.

[8] Engelmann B., Brautigam C., Kulschar R., Duhm J., Prenner E. (1994). Reversible reduction of phospholipid bound arachidonic acid after low density lipoprotein apheresis. Evidence for rapid incorporation of plasmalogen phosphatidylethanolamine into the red blood cell membrane. Biochim Biophys Acta.

[9] Weylandt K.H., Schmöcker C., Ostermann A.I., Kutzner L., Willenberg I. (2019). Activation of lipid mediator formation due to lipoprotein apheresis. Nutrients.

[10] Schmöcker C., Kassner U., Kiesler S., Bismarck M., Rothe M. (2016). A lipidomic analysis approach in patients undergoing lipoprotein apheresis. Atherosclerosis.

[11] Bolick D.T., Orr A.W., Whetzel A., Srinivasan S., Hatley M.E. (2005). 12/15-lipoxygenase regulates intercellular adhesion molecule-1 expression and monocyte adhesion to endothelium through activation of RhoA and nuclear factor-kappaB. Arterioscler Thromb Vasc Biol.

[12] Schmöcker C., Kassner U., Ostermann A.I., Kiesler S., Steinhagen-Thiessen E. (2017). Effect of different lipid apheresis methods on plasma polyunsaturated fatty acids. Atherosclerosis Suppl.

[13] Bhatt D.L., Steg P.G., Miller M., Brinton E.A., Jacobson T.A. (2019). Cardiovascular risk reduction with icosapent ethyl for hypertriglyceridemia. N Engl J Med.

[14] Yokoyama M., Origasa H., Matsuzaki M., Matsuzawa Y., Saito Y. (2007). Effects of eicosapentaenoic acid on major coronary events in hypercholesterolaemic patients (JELIS): a randomised open-label, blinded endpoint analysis. Lancet (London, England).

[15] Schunck W.H., Konkel A., Fischer R., Weylandt K.H. (2018). Therapeutic potential of omega-3 fatty acid-derived epoxyeicosanoids in cardiovascular and inflammatory diseases. Pharmacol Ther.

[16] Wang B., Wu L., Chen J., Dong L., Chen C. (2021). Metabolism pathways of arachidonic acids: mechanisms and potential therapeutic targets. Signal Transduct Targeted Ther.

[17] Kang J.X., Wang J. (2005). A simplified method for analysis of polyunsaturated fatty acids. BMC Biochem.

[18] Kutzner L., Rund K.M., Ostermann A.I., Hartung N.M., Galano J.M. (2019). Development of an optimized LC-MS method for the detection of specialized pro-resolving mediators in biological samples. Front Pharmacol.

[19] Hartung N.M., Mainka M., Pfaff R., Kuhn M., Biernacki S. (2023). Development of a quantitative proteomics approach for cyclooxygenases and lipoxygenases in parallel to quantitative oxylipin analysis allowing the comprehensive investigation of the arachidonic acid cascade. Anal Bioanal Chem.

[20] Rund K.M., Ostermann A.I., Kutzner L., Galano J.M., Oger C. (2018). Development of an LC-ESI(-)-MS/MS method for the simultaneous quantification of 35 isoprostanes and isofurans derived from the major n3- and n6-PUFAs. Anal Chim Acta.

[21] Stark K.D., Van Elswyk M.E., Higgins M.R., Weatherford C.A., Salem N. (2016). Global survey of the omega-3 fatty acids, docosahexaenoic acid and eicosapentaenoic acid in the blood stream of healthy adults. Prog Lipid Res.

[22] Calder P.C. (2013). Omega-3 polyunsaturated fatty acids and inflammatory processes: nutrition or pharmacology?. Br J Clin Pharmacol.

[23] Patterson E., Wall R., Fitzgerald G.F., Ross R.P., Stanton C. (2012). Health implications of high dietary omega-6 polyunsaturated Fatty acids. Journal of nutrition and metabolism.

[24] Bloomgarden Z.T. (2005). Inflammation, atherosclerosis, and aspects of insulin action. Diabetes Care.

[25] van Wijk D.F., Sjouke B., Figueroa A., Emami H., van der Valk F.M. (2014). Nonpharmacological lipoprotein apheresis reduces arterial inflammation in familial hypercholesterolemia. J Am Coll Cardiol.

[26] Wang Y., Blessing F., Walli A.K., Uberfuhr P., Fraunberger P. (2004). Effects of heparin-mediated extracorporeal low-density lipoprotein precipitation beyond lowering proatherogenic lipoproteins--reduction of circulating proinflammatory and procoagulatory markers. Atherosclerosis.

[27] Wieland E., Schettler V., Armstrong V.W. (2002). Highly effective reduction of C-reactive protein in patients with coronary heart disease by extracorporeal low density lipoprotein apheresis. Atherosclerosis.

[28] Rosada A., Kassner U., Vogt A., Willhauck M., Parhofer K. (2014). Does regular lipid apheresis in patients with isolated elevated lipoprotein(a) levels reduce the incidence of cardiovascular events?. Artif Organs.

[29] Gonzalez-Nunez D., Claria J., Rivera F., Poch E. (2001). Increased levels of 12(S)-HETE in patients with essential hypertension. Hypertension.

[30] Suzuki N., Hishinuma T., Saga T., Sato J., Toyota T. (2003). Determination of urinary 12(S)-hydroxyeicosatetraenoic acid by liquid chromatography-tandem mass spectrometry with column-switching technique: sex difference in healthy volunteers and patients with diabetes mellitus. J Chromatogr, B: Anal Technol Biomed Life Sci.

[31] Zhang H.J., Sun C.H., Kuang H.Y., Jiang X.Y., Liu H.L. (2013). 12S-hydroxyeicosatetraenoic acid levels link to coronary artery disease in Type 2 diabetic patients. J Endocrinol Invest.

[32] Szklenar M., Kalkowski J., Stangl V., Lorenz M., Ruhl R. (2013). Eicosanoids and docosanoids in plasma and aorta of healthy and atherosclerotic rabbits. J Vasc Res.

[33] Bojic L.A., McLaren D.G., Harms A.C., Hankemeier T., Dane A. (2016). Quantitative profiling of oxylipins in plasma and atherosclerotic plaques of hypercholesterolemic rabbits. Anal Bioanal Chem.

[34] Manega C.M., Fiorelli S., Porro B., Turnu L., Cavalca V. (2019). 12(S)-Hydroxyeicosatetraenoic acid downregulates monocyte-derived macrophage efferocytosis: new insights in atherosclerosis. Pharmacol Res.

[35] Zu L., Guo G., Zhou B., Gao W. (2016). Relationship between metabolites of arachidonic acid and prognosis in patients with acute coronary syndrome. Thromb Res.

[36] Reilly K.B., Srinivasan S., Hatley M.E., Patricia M.K., Lannigan J. (2004). 12/15-Lipoxygenase activity mediates inflammatory monocyte/endothelial interactions and atherosclerosis in vivo. J Biol Chem.

[37] Singh N.K., Rao G.N. (2019). Emerging role of 12/15-Lipoxygenase (ALOX15) in human pathologies. Prog Lipid Res.

[38] Wang X., Gao L., Xiao L., Yang L., Li W. (2019). 12(S)-hydroxyeicosatetraenoic acid impairs vascular endothelial permeability by altering adherens junction phosphorylation levels and affecting the binding and dissociation of its components in high glucose-induced vascular injury. Journal of Diabetes Investigation.

[39] Kühn H., Belkner J., Wiesner R., Schewe T., Lankin V.Z. (1992). Structure elucidation of oxygenated lipids in human atherosclerotic lesions. Eicosanoids.

[40] Kuhn H., Heydeck D., Hugou I., Gniwotta C. (1997). In vivo action of 15-lipoxygenase in early stages of human atherogenesis. J Clin Invest.

[41] Waddington E., Sienuarine K., Puddey I., Croft K. (2001). Identification and quantitation of unique fatty acid oxidation products in human atherosclerotic plaque using high-performance liquid chromatography. Anal Biochem.

[42] Vangaveti V., Baune B.T., Kennedy R.L. (2010). Hydroxyoctadecadienoic acids: novel regulators of macrophage differentiation and atherogenesis. Ther Adv Endocrinol Metab.

