# Supplementary material for: Changing from lipoprotein apheresis to evolocumab treatment lowers circulating levels of arachidonic acid and oxylipins
Source: Atheroscler Plus. 2024 Feb 12;55:55–62. doi: 10.1016/j.athplu.2024.01.005 (PMC10881432; doi:10.1016/j.athplu.2024.01.005)
Supplement: Multimedia component 1 [file mmc1.docx]

**Supplementary Figure S1** Change of lipid profile parameters of patients receiving lipoprotein apheresis or evolocumab after six weeks of treatment. Shown are the mean ratios of the lipid parameters at week 6 to the baseline concentration at day 1 of (A) lipoprotein(a), total cholesterol and triglycerides, (B) low-density lipoprotein (LDL), high-density lipoprotein (HDL) and very low-density lipoprotein (VLDL) cholesterol (n = 19 for lipoprotein apheresis, n = 18 for evolocumab). Values are presented as mean + SEM. Statistical analyses were performed by two-tailed unpaired Student’s *t*-test. *P* < 0.0083 after Bonferroni correction is considered statistically significant.

**Supplementary Figure S2** Fatty acid profile in the serum of patients receiving lipoprotein apheresis or evolocumab. Shown are the concentrations after six weeks of treatment of (A) SFA, MUFA and PUFA, (B) n-3 and n-6 PUFAs, (C) individual n-3 PUFAs EPA, DPA and DHA, and (D) individual n-6 PUFAs LA, DGLA, AA and AdA (n = 19 for lipoprotein apheresis, n = 16 for evolocumab). Values are presented as mean + SEM. Statistical analyses were performed by two-tailed unpaired Student’s *t*-test. *P* < 0.0042 after Bonferroni correction is considered statistically significant.

**Supplementary Figure S3** Oxylipin levels in the plasma of patients receiving lipoprotein apheresis or evolocumab. Shown are the concentrations after six weeks of treatment of selected monohydroxy fatty acids derived from (A) LA, (B) AA, (C) EPA, and (D) DHA. Values are presented as mean + SEM (n = 19 for lipoprotein apheresis, n = 16 for evolocumab). Statistical analyses were performed by two-tailed unpaired Student’s *t*-test. *P*< 0.0039 after Bonferroni correction is considered statistically significant. LLOQ, lower limit of quantification
