# Supplementary material for: Changing from lipoprotein apheresis to evolocumab treatment lowers circulating levels of arachidonic acid and oxylipins
Source: Atheroscler Plus. 2024 Feb 12;55:55–62. doi: 10.1016/j.athplu.2024.01.005 (PMC10881432; doi:10.1016/j.athplu.2024.01.005)
Supplement: Multimedia component 2 [file mmc2.docx]

**Supplementary Table S1** Baseline characteristics at day 1

| **Characteristic** | **Lipoprotein apheresis (n = 19)** | **Evolocumab**  **(n = 18)** | ***p* value** | **Total**  **(n = 37)** |
| --- | --- | --- | --- | --- |
| Mean age (SD), y | 59.8 (10.3) | 65.1 (8.2) |  | 62.4 (9.6) |
| Male, n (%) | 12 (63.2) | 10 (55.6) |  | 22 (59.5) |
| Race |  |  |  |  |
| White, n (%) | 19 (100.0) | 17 (94.4) |  | 36 (97.3) |
| Black or African American, n (%) | 0 | 1 (5.6) |  | 1 (2.7) |
| Lipid levels |  |  |  |  |
| Cholesterol, mean (SD), mmol/L | 6.03 (0.88) | 6.18 (0.85) | 0.6138 | 6.10 (0.86) |
| LDL-C, mean (SD), mmol/L | 3.96 (0.75) | 3.99 (0.64) | 0.8771 | 3.98 (0.69) |
| HDL-C, mean (SD), mmol/L | 1.17 (0.30) | 1.23 (0.34) | 0.5956 | 1.20 (0.32) |
| VLDL-C, mean (SD), mmol/L | 0.78 (0.30) | 0.87 (0.43) | 0.4836 | 0.82 (0.37) |
| Triglycerides, mean (SD), mmol/L | 1.91 (1.12) | 2.05 (1.13) | 0.6906 | 1.98 (1.12) |
| Lp(a), mean (SD), mmol/L | 58.11 (67.75) | 115.33 (103.46) | 0.0532 | 85.95 (90.50) |
| Fatty acid concentrations |  |  |  |  |
| Total, mean (SD), µg/µl | 2.18 (0.37) | 2.25 (0.48) | 0.6029 | 2.21 (0.42) |
| SFA, mean (SD), µg/µl | 0.90 (0.16) | 0.92 (0.18) | 0.6256 | 0.91 (0.17) |
| MUFA, mean (SD), µg/µl | 0.42 (0.14) | 0.46 (0.18) | 0.4476 | 0.44 (0.16) |
| PUFA, mean (SD), µg/µl | 0.86 (0.15) | 0.87 (0.16) | 0.9166 | 0.87 (0.15) |
| n-3 PUFA, mean (SD), µg/µl | 0.14 (0.04) | 0.15 (0.05) | 0.4193 | 0.14 (0.05) |
| n-6 PUFA, mean (SD), µg/µl | 0.73 (0.14) | 0.72 (0.16) | 0.8834 | 0.72 (0.14) |

HDL-C, high-density lipoprotein cholesterol; LDL-C, low-density lipoprotein cholesterol; Lp(a), lipoprotein(a); MUFA, monounsaturated fatty acid; n-3/n-6 PUFA, omega-3/6 PUFA; PUFA, polyunsaturated fatty acid; SFA, saturated fatty acid; VLDL-C, very low-density lipoprotein cholesterol.

Statistical analyses were performed by two-tailed unpaired Student’s *t*-test.
